# Forearmed is forewarned: A prospective intervention observational time‐series study of patient empowerment for ultrasound‐guided peripheral intravenous access

**DOI:** 10.1111/1742-6723.13981

**Published:** 2022-05-17

**Authors:** Eng Libbis, Amy L Sweeny, Travis Holmes, Nisha Aggarwal, Peter J Snelling, Eugene Slaughter, Hugo Poncia, Stuart C Watkins

**Affiliations:** ^1^ Emergency Department Gold Coast Hospital and Health Service Gold Coast Queensland Australia; ^2^ School of Medicine and Dentistry Griffith University Gold Coast Queensland Australia; ^3^ Alliance for Vascular Access Teaching and Research Schools of Nursing and Midwifery, and Pharmacy and Medical Sciences, Griffith University Gold Coast Queensland Australia; ^4^ Faculty of Health Sciences and Medicine Bond University Gold Coast Queensland Australia; ^5^ Child Health Research Centre The University of Queensland Brisbane Queensland Australia; ^6^ School of Nursing Midwifery and Social Work, The University of Queensland Brisbane Queensland Australia; ^7^ Emergency Department The Tweed Hospital Tweed Heads New South Wales Australia

**Keywords:** patient advocacy, patient empowerment, peripheral intravenous cannula, ultrasound, ultrasound guided

## Abstract

**Objective:**

Ultrasound (US) is a valuable adjunct to improve the success rates of difficult peripheral intravenous cannula (PIVC) insertions but is usually clinician initiated. The present study assessed for any change in clinician practice resulting from interventions aimed at empowering patients to advocate for early use of US if they self‐identified as having difficult PIVC access.

**Methods:**

This was a prospective observational time‐series study using a rapid quality improvement (RQI) framework. Three ED waiting room intervention strategies (printed media, video and wristband) were tested over three 2‐week periods at a large teaching hospital. The impact of each intervention was assessed at eight time points during each intervention and compared to a pre‐intervention baseline period using trend and time‐series analysis.

**Results:**

A total of 1611 PIVC insertions were surveyed over 42 time points. The proportion of US‐guided PIVC insertions was highest during Intervention 3 (wristbands; 5.5%) but all proportions remained below baseline (6.5%). Trend analysis identified an increasing frequency of US use during Intervention 1 (printed media, *P* = 0.01). However, no statistically significant trends were observed within the periods.

**Conclusions:**

This is the first prospective study to assess the effect of various interventions to empower patients to self‐identify as having difficult PIVC access and advocate for the use of US‐guidance. The present study was indeterminate: no intervention tested in the present study noticeably influenced clinical practice, potentially attributable to the study design and confounding factors. This innovative study serves as a pilot for future research into patient empowerment, which is currently lacking in the literature.

## Graphical abstract

Ultrasound (US) is a valuable adjunct to improve the success rates of difficult PIVC insertions but is usually clinician initiated. Three ED waiting room interventions were tested to determine their effect on empowering patients to advocate for early use of US if they self‐identified as having difficult PIVC access. Although no single intervention decidedly changed the proportion of PIVC placed with US‐guidance, the concept and time‐efficient, resource‐efficient design of this study is read‐worthy.

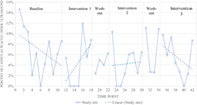

**Key findings**

- This study tests three single interventions to improve the uptake of US during cannulation of difficult to cannulate patients.
- Although no single intervention decidedly changed the proportion of PIVC placed with US‐guidance, the concept and time‐efficient, resource‐efficient design of this study informs further research in this area.

## Introduction

Peripheral intravenous cannula (PIVC) insertion is one of the most common invasive medical interventions performed in the ED, with up to two‐thirds of patients requiring PIVC access during hospitalisation.^1^, ^2^ A recent study found that around 10% of patients endure three or more PIVC insertion attempts before success, which poses concerns of cost, time and patient wellbeing.^1^, ^3^ Multiple PIVC insertion attempts in these patients with difficult intravenous access (DIVAs) has been associated with complications, including insertion site pain, phlebitis, extravasation, occlusion and infection (local and systemic).^4^, ^5^ Furthermore, the fear of failed cannulation attempts may cause delayed patient presentation or treatment refusal.^5^ Up to half of all adult patients have one or more DIVA risk factors, with the most common being having no visible or palpable vein, and/or having a known history of difficult cannulation.^5^, ^6^

Ultrasound (US)‐guided PIVC insertion is known to increase first pass success in patients assessed as DIVA.^7^, ^8^ Despite this, US guidance is typically only employed after several failed PIVC insertion attempts.^3^, ^6^, ^7^ A 2018 meta‐analysis by Van Loon *et al*. evaluated eight studies with a total of 1660 patients, demonstrating that US guidance increased patient satisfaction and reduced the number of PIVC attempts.^8^ Similarly, a literature review by Sabri *et al*. evaluated 128 papers, demonstrating that the first attempt of PIVC insertion, in the absence of US guidance, failed in 12–26% of adult cases. The use of US as an adjunct not only reduced the number of attempts, but also reduced procedure duration while improving overall patient satisfaction.^7^ Moreover, US guidance has been reported to increase PIVC insertion success rates threefold compared to traditional techniques.^9^ However, studies to date emphasise its implementation by educating the clinician, rather than empowering the patient.

The present study aimed to assess the change in clinician practice resulting from a variety of interventions empowering self‐identified DIVA patients to advocate for the use of US guidance, if they required PIVC insertion.

## Methods

### Study design

This was a prospective observational time‐series study designed using the theoretical framework of rapid quality improvement (RQI).^10^ The following attributes of the question we wanted to answer lent itself to this design:^10^, ^11^ (i) we were measuring the effect of an intervention over time; (ii) subsequent observations were not independent of each other; and (iii) we wanted to test several interventions briefly.

### Key outcomes

The primary outcome was the change in the rate of US‐guided PIVC insertions per 100 ED cannulations for each intervention period, compared to baseline.

Secondary outcomes were (i) the number of landmark PIVC insertion attempts before the use of US guidance; and (ii) total PIVC insertions as a proportion of all presentations in the ED acute care setting.

### Study setting and population

The study was conducted over a 3‐month period (August–November 2019) within the ED of a tertiary teaching hospital in Southeast Queensland (study site).

The study site is an academic hospital with Level 3 trauma services that manages over 113 000 presentations annually.^12^ Approximately 345 full‐time equivalent nurses and doctors work in the study site's ED. A recent study undertaken at this site established the prevalence of DIVA adult patients of 35%.^6^ An emergency US training programme, incorporating vascular access is embedded in the study site.^13^

Patient eligibility criteria included: adult aged 18 years and over, English‐speaking, present in an acute care area of ED (short stay and mental health areas excluded) and required PIVC‐placement by ED staff. Patients with PIVC inserted pre‐hospital were excluded. It was assumed that the proportion of patients with DIVA remained constant over the study period and, therefore, was not measured.

### Study protocol and data collection

**Figure 1:**
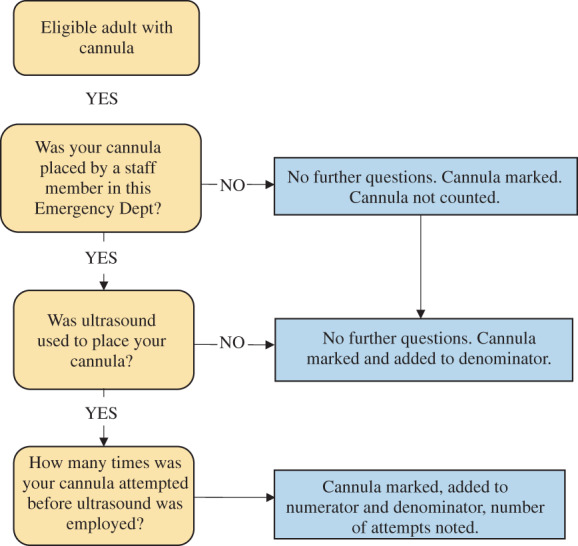
Study procedure for data capture of cannulas placed by ED staff and concurrent use of ultrasound.

A pragmatic data collection strategy was used (Fig. 1) over a series of 4 h periods, which tallied both the number of eligible patients (denominator), and the outcomes of interest (numerator[s]), without requiring documentation of any personal details.

Data collection was performed by research members who were not on clinical shifts. The location of PIVC placement (in ED or pre‐hospital) was ascertained by asking the patient; then a sticker was placed on their PIVC dressing to indicate their inclusion if eligible. Data collectors returned to ED at the middle and end of the 4 h period to recruit any additional eligible patients. At the control site, data were collected under the same design on the same days during intervention periods.

**Figure 2:**
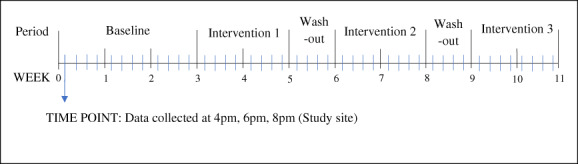
Visual representation of the study design. Blue lines represent time points which constitute the 4 h window in which data were collected.

Data collection was structured across six periods: an initial non‐interventional 3‐week period to ascertain baseline data, followed by three intervention periods, each of 2‐week duration, separated by a 1‐week wash‐out interval (Fig. 2). Following the minimum recommendations for time‐series analysis,^14^ data were to be collected at eight time points during the baseline and each intervention phase.

Throughout each baseline or intervention period, data were collected on 8 or more separate days during a 4 h window each day (16.00–18.00 hours). Each 4 h window was considered a single time point (Fig. 2). This time window reflected one of the busiest times of the day at the study site.

Data on the number of ED presentations managed in the acute care areas during each 4 h observation window, and the number within each Australasian Triage Score (ATS) category 3–5, was abstracted retrospectively from the electronic patient tracking system.

### Interventions

Three different interventions were utilised during the present study. Each intervention consisted of different media, inviting patients to alert their clinician of difficult cannulation experiences in the past, and to increase the early utilisation of US guidance by appropriately trained providers. The four arms of the study were baseline data and three interventions: (i) printed media (posters and flyers); (ii) visual media (a video on display in the waiting room); and (iii) visual awareness media using specific wristbands on patients identified as previously difficult.

#### Intervention 1

Twelve single‐sided A3‐sized posters and A5‐sized flyers were showcased strategically throughout study site's emergency waiting room, triage bays and ambulance ramp. These listed risk factors for difficult PIVC insertion and invited self‐identified patients to ask about the use of US (Appendix S1). These remained in place for the entirety of the 2‐week intervention period.

#### Intervention 2

The same information as Intervention 1 was played in video format every hour during the intervention period on two large wall‐mounted televisions in the ED waiting room. The video^15^ expanded on cannulation practices in more detail and was played with subtitles without audio because of high ambient noise of an ED waiting room.

#### Intervention 3

A yellow wristband with the wording ‘HX OF DIFFICULT IV ACCESS’ (Appendix S2) was placed on self‐identified DIVA patients. This intervention was driven by the triage nurses, who asked each adult patient presenting during each 4 h study period if they had a history of difficult PIVC insertion. The wristband colour was chosen to be distinct from the usual white or red patient identification and allergy wristbands.

Each of the intervention strategies was removed on commencement of the washout periods to avoid cross‐over effects of each intervention.

### Data analysis

The primary outcome (change in the rate of US‐guided PIVC insertions per 100 PIVCs placed in ED) was assessed using: (i) linear regression trend analysis for each period (SPSS v26.0), calculating the running slope difference *t* test;^16^ and (ii) traditional time‐series analysis in R (v3.6.0).^17^ For the trend analysis, each intervention period was compared to the baseline period. Simple descriptive statistics (counts and proportions) were calculated, along with mean rates and 95% confidence intervals (CIs) for the mean for each period of interest.

In R, an ARIMA time‐series model was built depicting all observation periods (baseline, Interventions 1–3 and washout periods 1 and 2). Autocorrelation was assessed by plotting the autocorrelation factor against the lagged result. Non‐stationarity was assessed using the augmented Dickey–Fuller test. It was assumed that seasonality did not need consideration since the time‐series spanned only 10 weeks, and that the proportion of staff trained in US‐guided PIVC access remained static throughout the time periods.

Secondary outcomes were assessed with the *χ* ^2^‐test using Fisher's exact result.

### Ethics

The present study received a waiver of review from the Gold Coast Hospital and Health Service Human Research Ethics Committee (HREC Reference LNR/2021/QGC/73370).

## Results

Over the 3‐month period, data were collected at 42 time points; 1611 PIVC insertions were included at the study site, with 87 inserted under US guidance (US‐guided proportion [95% CI] 5.4% [4.4–6.6%]). The cumulative results of the study's primary outcome measure are listed in Table 1 by period.

**TABLE 1:** Use of ultrasound (US) during peripheral intravenous cannula (PIVC) placement in the ED: use per 100 PIVCs and 95% confidence intervals (CIs) around the proportion

Period	Total	US used		3+ attempts prior to US	
	PIVCs	*n*	% (95% CI)	*n*	% (95% CI)
Baseline	428	28	6.5 (4.6–9.3)	4	14.3 (5.7–31.5)
Intervention 1	259	12	4.6 (2.7–7.9)	6	50.0 (25.4–74.6)
Intervention 2	299	12	4.0 (2.3–6.9)	3	25.0 (8.9–53.2)
Intervention 3	329	18	5.5 (3.5–8.5)	3	16.7 (5.8–39.2)
Overall†	1611	87	5.4 (4.4–6.6)	20	23.0 (15.4–32.9)

† Includes data collected during the two wash‐out periods at the study site, not displayed here.

Overall, it was not observed that any of the interventions had a noticeable impact on changing the rates of US‐guided PIVC insertions or that they decreased the number of patients requiring more than three PIVC insertion attempts.

With respect to the primary outcome, it was observed that the proportion of US‐guided PIVC insertions was highest during Intervention 3 (wristbands; 5.5%), but all proportions during the intervention phases were below that of baseline (6.5%). This finding raised concern that there may have been a disproportionate number of doctors considered to be US advocates and experts in PIVC placement rostered on during baseline shifts. However, a review of the rosters from the study period determined that the presence of US advocates did not affect the proportion of patients who had US‐guided PIVCs. First, the proportion of PIVCs placed with US was 5.9% across the 13 shifts with a US advocate, compared to 6.1% across the 21 shifts without. Second, the Pearson's correlation coefficient of US‐guided PIVC rate and the number of advocate(s) rostered was small 0.037.

Regarding the secondary outcomes, 23% of patients endured three or more attempts of PIVC insertion prior to use of US at the study site. This proportion was lowest during Intervention 3, but all proportions during the intervention periods were higher than that of baseline. Differences in this proportion from baseline to each of the intervention periods were not statistically significant. Because of the small number of PIVCs placed under US, time‐series and trend analyses were not conducted on this secondary outcome (Table 1).

**Figure 3:**
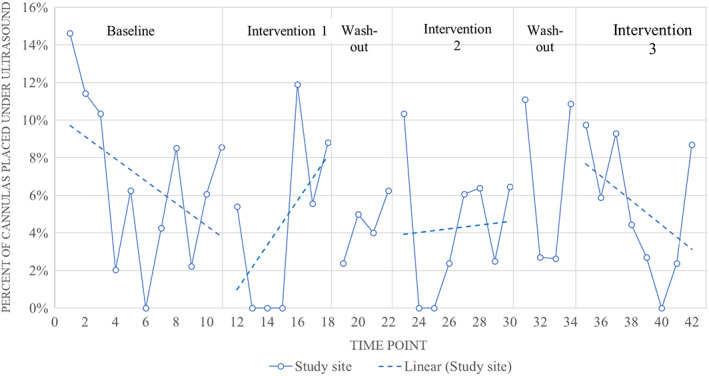
Proportion of peripheral intravenous vascular cannula placed using ultrasound guidance by time point for each time in the study site and control site EDs. Dotted lines indicate overall trend for each intervention at study site.

Trend analysis identified a statistically significant and beneficial change in the US rate trend during Intervention 1 (*P* = 0.01), suggesting an increasing frequency of US use (Table 2, Fig. 3).

**TABLE 2:** Trend statistics for baseline and intervention periods when fitted to linear regression model

Periods	Observation periods	Slope	Standard error (slope)	*t*‐statistic	*P*‐value	Interpretation	
Baseline	11	−0.593	0.402	−1.475	0.174	No statistically significant trend within the period	
Intervention 1	7	1.187	0.831	1.429	0.212	No statistically significant trend within the period	
Intervention 2	8	0.096	0.604	0.159	0.879	No statistically significant trend within the period	
Intervention 3	8	−0.65	0.54	−1.198	0.276	No statistically significant trend within the period	
*Change in slope, intervention to baseline*							
Periods	Degrees of freedom	Change in slope		RSD *t*‐statistic	*P*‐value	Interpretation	
Baseline to Intervention 1	14	1.78		2.927	0.011	Statistically significant change in trend from baseline	
Baseline to Intervention 2	15	0.689		1.236	0.235	No statistically significant change in trend from baseline	
Baseline to Intervention 3	15	−0.057		−0.106	0.917	No statistically significant change in trend from baseline	

RSD, running slope difference.

The rate of PIVC insertions by ED staff for all eligible ED presentations during data collection periods at the study site was 38 per 100 adult presentations to acute care areas (Table 3). This rate decreased significantly from baseline (42.6 per 100) to 36.1 during Intervention 1 (*P* < 0.01) and 35.0 per 100 during Intervention 2 (*P* < 0.001).

**TABLE 3:** Characteristics considered to be potentially confounding factors for ultrasound‐guided cannulation rates, captured for baseline and intervention 4 h observation periods at the study site

Period	Total triaged	Total ATS 1 or 2	Total cannulations	Cannulation rate per 100 triaged	ATS 1 or 2 per 100 triaged
Baseline	1004	268	428	42.6	26.7
Intervention 1	718	194	259	36.1*	27.0
Intervention 2	855	247	299	35.0**	28.9
Intervention 3	843	202	329	39.0	24.0
Overall	4201	1125	1611	38.3	26.8

*P < 0.01. **P < 0.001 for period compared to baseline. ATS, Australasian Triage Score.

## Discussion

Patient empowerment is an important facet of clinical care. Over the past few decades, there has been a paradigm shift from medical paternalism to increase patient‐centred care, based on a biopsychosocial model.^18^ Therefore, it is imperative to explore holistic strategies in healthcare that empowers patients to advocate for their own health. Given its numerous benefits, US‐guided PIVC insertion has become the standard of care in the management of patients with DIVA^19^ but is still poorly adopted by clinicians. Therefore, patients who self‐identify as DIVA should be empowered to request its use by an appropriately trained clinician. The present study, although indeterminate, juxtaposes two highly relevant areas of medicine, addressing an important gap in the literature of which, to our knowledge, no studies to date have specifically evaluated.

There have been a small number of studies that have assessed patient empowerment in the acute care setting and the impact that it has on a patient's healthcare journey.^20^, ^21^ A study by McDonall *et al*. examined whether the use of multimedia interventions, such as paper‐based materials, posters and video instructions, improved a patient's knowledge in their acute care, leading to increased quality and safety of the care they received.^20^ The narrative review of 10 papers found that there was high patient satisfaction with use of the multimedia interventions, showing feasibility in the use of the materials, but further studies would be needed to evaluate whether there were increased instances of improved patient‐centred outcomes. A study by McGuckin and Goednik showed that patients are willing to be empowered in the healthcare setting.^21^

Our study utilised an RQI framework incorporating three methods in small‐scale testing to evaluate the effect of patient‐targeted interventions on US‐guided PIVC insertion. There appeared to be a statistically significant change in practice with the use of US from the baseline period to the first intervention, but the authors suggest that this should be interpreted cautiously.

Although it is not attributed solely to its aggregate number of data points, a time‐series should include a minimum of eight points to infer a trend for a specific period of time.^11^, ^14^ This was a driving force in the design of the present study and was adhered to throughout the periods of intervention with the exception of Intervention 1, in which only seven data points could be recorded. It is possible that by extending the time frame for each period and increasing the total number of data points, a more stable trend could be inferred from the dataset. The power of series is also enhanced with equal distribution of data points prior to and following an intervention,^11^ which constitutes another consideration when optimising further research.

An unexpected finding of our study was a decrease in the overall ED‐initiated PIVC rate observed during two of the three intervention periods. This phenomenon could have been influenced by practitioner behaviour and the proportion of patients requiring PIVC insertion during the study period. Practitioner behaviour could have been influenced by the open‐label methods of the trial, leading to less PIVC insertions in patients. Future studies should account for a fluctuating overall PIVC rate, as this is potentially a confounding factor.

### Limitations

There were several limitations to the present study. First, the present study did not control for the number of US‐trained clinicians and/or US advocates rostered on each shift. This was, however, unlikely to bias our findings, as described in Results section. Nonetheless, the presence or absence of specialist staffing across a small number of study periods could be an incurable confounder for future studies, and should be part of future study designs.

Second, fidelity for each intervention was not assessed, and therefore it is difficult to determine whether interventions were effectively deployed, reaching and saturating the intended audience. The passive interventions used in Intervention 1 (posters and flyers) and Intervention 2 (video) were likely missed by some patients in the waiting rooms. Patients who were brought straight into any area of the ED by ambulance would not have seen the video as it was only being played in the waiting room. The video itself was played without sound, which may limit its audience. During Intervention 3, it is uncertain if all patients were asked the appropriate questions by the triage team and whether the wristbands were offered to or accepted by the patients. In retrospect, assessing fidelity to each intervention would improve the present study's interpretation. Clarifying with each patient during data collection if they were aware of the respective intervention would help ascertain if there were limitations surrounding visibility of the intervention or with the intervention strategy itself. The data collectors were not blinded to the study's aims or methodology, which could have led to recorder bias as well.

Each intervention period ran for only 2 weeks, with eight data collection blocks because of the condensed time frame of availability for data collection. The time of data collection only captured a small portion of the day. Designing a further study with longer time periods for each of the interventions might be beneficial to capture a more accurate rate of patients with DIVA and use of US guidance.

Finally, the single‐site setting limits generalisability. Extrapolating the present study over a longer period at multiple sites may allow for cannulation trends to be better identified, including the differentiation between external influences and normal fluctuations.

#### Additional recommendations for future research

The choice to place a PIVC, and how to do so, is a complex and common decision ED clinicians must make on a daily basis. The intervention involves the patient's characteristics, the urgency of their condition, the clinician and his/her skillset, the culture of the ED and equipment available, among other factors. Allowing the patient to have some control over if – and how – this is done is vital to increasing patient‐centred care. The present study attempted to influence one aspect of the intervention – the consumer. Undoubtedly, cannulation of a DIVA patient is a complex intervention, and therefore would likely benefit from a multi‐factorial research approach that combines strategies aimed at the environment, the clinician and the consumer.^22^

## Conclusions

This is the first prospective observational study aiming to empower the patient, rather than the clinician, to advocate for US‐guided PIVC insertion. Although the present study was indeterminate, it populates a profound gap in the literature and serves as a scaffold for future research. We recommend that subsequent studies of this type should account for potential confounders, such as staffing and PIVC insertion rates, measure intervention fidelity and extend the duration of intervention and wash‐out periods, or randomise multiple different sites to the intervention, to truly evaluate the impact of patient empowerment on US‐guided PIVC insertion within the ED.

## Supporting information


**Appendix S1**. Example of poster/flyer used in Intervention 1.

**Appendix S2**. Example of wristband used in Intervention 3.

## Data Availability

Data are available upon reasonable request to the corresponding author.
