# Supplementary material for: Forearmed is forewarned: A prospective intervention observational time‐series study of patient empowerment for ultrasound‐guided peripheral intravenous access
Source: Emerg Med Australas. 2022 May 17;34(5):779–85. doi: 10.1111/1742-6723.13981 (PMC9790456; doi:10.1111/1742-6723.13981)
Supplement: Supplementary file 1 — Appendix S1. Example of poster/flyer used in Intervention 1. [file EMM-34-779-s002.docx]

**Appendix S1**

Example of poster/flyer using in Intervention 1
