# Supplementary figures and images for: Forearmed is forewarned: A prospective intervention observational time‐series study of patient empowerment for ultrasound‐guided peripheral intravenous access
Source: Emerg Med Australas. 2022 May 17;34(5):779–85. doi: 10.1111/1742-6723.13981 (PMC9790456; doi:10.1111/1742-6723.13981)

**Appendix S2**

Example of wristband used in Intervention 3


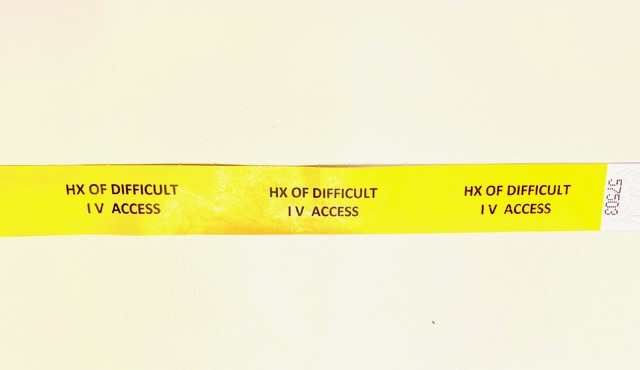

Supplement: Supplementary file 2 — Appendix S2. Example of wristband used in Intervention 3. [file EMM-34-779-s001.docx]
